# Supplementary material for: Effect of acipimox on skeletal muscle biochemistry, structure and function in older people with probable sarcopenia: an experimental medicine study
Source: GeroScience. 2025 Mar 18;47(4):6065–78. doi: 10.1007/s11357-025-01606-9 (PMC12397013; doi:10.1007/s11357-025-01606-9)
Supplement: Supplementary file 1 — Supplementary file1 (DOCX 38 KB) [file 11357_2025_1606_MOESM1_ESM.docx]

# **Supplementary Data**

**Supplementary Table 1: Eligibility Criteria**

| **Eligibility Criteria**    *Inclusion Criteria*  ALL of the following inclusion criteria must be met:  - Age 65 years or over  - Low maximum handgrip strength (<16kg for women, <27kg for men) OR five-times sit-to-stand time >15 seconds (inability to complete sit-to-stand test will count as time > 15 seconds)  - Walk speed <=0.8 m/s on 4 metre walk test  *Exclusion Criteria*  General:  - Allergy to Acipimox or other niacin-related products  - Allergy or intolerance of aspirin  - Any contraindication to taking aspirin  - Unable to give written informed consent  - Currently enrolled in another intervention study (observational studies are permitted)  - Currently participating in supervised exercise classes or physiotherapy  - Any progressive neurological or malignant condition with a life expectancy <6 months  Safety of IMP and nIMP:  - Creatinine Clearance <45ml/min (by Cockcroft-Gault equation)  - Taking statin medication or fibrate medication  - Active peptic ulcer disease or dyspepsia  Safety of muscle biopsy and MRI:  - Platelets <100x109/L at screening (contraindication to muscle biopsy)  - Presence of a bleeding diathesis or use of oral or parenteral anticoagulant medication  - Antiplatelet agents other than low dose (75mg once daily) aspirin  - Contraindications to MRI scanning (mild claustrophobia is not a contraindication)  - Allergy to local anaesthetic (lidocaine)  - Unable to palpate vastus lateralis muscle to enable biopsy localisation  Other causes of skeletal myopathy:  - Liver function tests (bilirubin, ALT, alkaline phosphatase) > 3x ULN  - Symptomatic (NYHA class II-IV) chronic heart failure (diagnosed according to European Society of Cardiology guidelines)  - Severe COPD (GOLD stage IV)  - Known myositis or other established myopathy  - Self-reported weight loss of >10% in the last six months (to exclude significant cachexia)  - Known uncontrolled thyrotoxicosis  - 7.5mg/day or greater prednisolone use (or equivalent) |
| --- |

**Supplementary Table 2: Reported Exclusion criteria during the screening process.**

| **Reported Exclusion Criteria (some participants reported more than one exclusion criterion)**   - Progressive neurological or malignant condition with life expectancy <6 months (N=2) - Symptomatic (NYHA class II-IV) chronic heart failure (N=5) - Severe COPD (GOLD stage IV) (N=1) - 10% weight loss (N=5) - Taking statin medication or fibrate medication (N=9) - Use of oral or parenteral anticoagulant medication (N=5) - Antiplatelet agents other than low-dose aspirin (N=2) - Allergy to aspirin (N=1) - Contraindications to MRI scanning (N=2) - Corticosteroid equivalent to >7.5mg oral prednisolone (N=2) |
| --- |

**Supplementary Table 3: Spearman's correlations between blood NAD / NADH and muscle NAD / NADH.**

| **Baseline** | Rho (p) |
| --- | --- |
| Whole blood NAD vs Skeletal muscle NAD | -0.08 (p=0.83) |
| Whole blood NADH vs Skeletal muscle NADH | -0.67 (p=0.05) |
| Whole blood NAD/NADH ratio vs Skeletal muscle NAD/NADH ratio | -0.50 (p=0.17) |
| **Follow up** |  |
| Whole blood NAD vs Skeletal muscle NAD | 0.19 (p=0.65) |
| Whole blood NADH vs Skeletal muscle NADH | -0.57 (p=0.18) |
| Whole blood NAD/NADH ratio vs Skeletal muscle NAD/NADH ratio | 0.07 (P=0.88) |

**Supplementary Table 4: Summary of adverse event frequencies**

|  | | Number of participants (N=11) |
| --- | --- | --- |
| Number of participants with at least one AE | | 9 |
| Number of participants with at least one AR | | 5 |
| Number of participants with at least one SAE | | 0 |
| Number of participants with at least one SAR | | 0 |
| Number of participants with at least one SUSAR | | 0 |
|  | |  |
| Total number of AEs | | 19 |
| Number of the most common AEs: | Itch | 1 |
|  | Soft tissue injury to the leg | 1 |
|  | Flushing | 1 |
|  | Cellulitis | 2 |
|  | Covid 19 Infection | 1 |
|  | Hematoma | 2 |
|  | Aching legs | 1 |
|  | Rash | 1 |
|  | Bruising to arm | 1 |
|  | Sciatica | 1 |
|  | Fall | 1 |
|  | Constipation | 1 |
|  | White coat hypertension | 1 |
| Number of hospital admissions | | 0 |
| Number of deaths | | 0 |

AE: Adverse even
