# Supplementary material for: Effect of acipimox on skeletal muscle biochemistry, structure and function in older people with probable sarcopenia: an experimental medicine study
Source: GeroScience. 2025 Mar 18;47(4):6065–78. doi: 10.1007/s11357-025-01606-9 (PMC12397013; doi:10.1007/s11357-025-01606-9)
Supplement: Supplementary file 2 — Supplementary file2 (DOCX 219 KB) [file 11357_2025_1606_MOESM2_ESM.docx]

**Acipimox to improve muscle function and sarcopenia – a feasibility study**

Statistical Analysis Plan

SAP Version number: 1.0

SAP Date: 25/04/2024

This statistical analysis plan is based on protocol version 2 [13/12/2021]

ISRCTN Number: 87404878

EudraCT Number: 2021-000993-28

REC Reference: 21/NE/0100

Sponsor: Newcastle upon Tyne Hospitals NHS Foundation Trust

Sponsor protocol number: 09768

Funder: MRC Confidence in Concept (Newcastle)

Funder reference number: NU-005871

**Prepared by:**

| Name | Claire McDonald | Role | Deputy Chief Investigator |
| --- | --- | --- | --- |
| Signature | 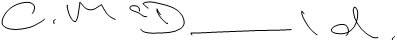 | Date | 26/04/2024 |

**Authorised by:**

| Name | Miles Witham | Role | Chief Investigator |
| --- | --- | --- | --- |
| Signature | 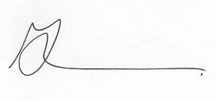 | Date | 26^th^ April 2024 |

This current version of the SAP and all preceding versions will be stored in the Statistical Section of the Trial Master File

This statistical analysis plan (SAP) provides a framework and guidelines for the statistical analysis and reporting of the *Acipimox to improve muscle function and sarcopenia – a feasibility study*

The SAP applies to a clean and validated dataset. Detailed information on data collection tools, data validation, consistency and accuracy checks and data storage and archiving can be found in the current versions of the Data Management Plan (version 1.0 [07/07/22])

Any deviation from the methods outlined in this SAP will be documented in the statistical end of trial report. Example Tables, Figures and Listings are for illustrative purposes only and are subject to change.

**Revision history**

| **Version** | **Date** | **Changes made** | **Justification for change** | **Timing of change** |
| --- | --- | --- | --- | --- |
| 1 | 26/04/2024 |  |  |  |
|  |  |  |  |  |
|  |  |  |  |  |
|  |  |  |  |  |
|  |  |  |  |  |
|  |  |  |  |  |
|  |  |  |  |  |
|  |  |  |  |  |

**Abbreviations**

| ADP | Adenosine diphosphate |
| --- | --- |
| AE | Adverse event |
| ALT | alanine transaminase |
| ANOVA | Analysis of the variance |
| ATP | Adenosine triphosphate |
| BD | Twice a day |
| CI | Chief investigator |
| CKD | Chronic kidney disease |
| COPD | Chronic obstructive pulmonary disease |
| eGFR | Estimated glomerular filtration rate |
| FBC | Full blood count |
| IMP | Investigational Medicinal Product |
| IQR | Interquartile range |
| ITT | Intention to treat |
| MRI | Magnetic resonance imaging |
| MRS | Magnetic resonance spectroscopy |
| mtDNA | Mitochondrial Deoxyribonucleic acid |
| NAD | Nicotinamide adenine dinucleotide |
| NAD(P)H | Nicotinamide adenine dinucleotide phosphate |
| NADH | Reduced NAD |
| NYHA | New York Heart Association |
| PCR | Polymerase chain reaction |
| PCr | Phosphocreatine |
| PIC | Participant identification centre |
| PP | Per protocol |
| SAE | Serious adverse event |
| SAP | Statistical analysis plan |
| SAR | Serious adverse reaction |
| SARC F | Strength, assistance with walking, rising from a chair, climbing stairs, and falls questionnaire |
| SD | Standard deviation |
| SOC | System organ class of concern |
| SPPB | Short physical performance battery |
| SUSAR | Suspected Unexpected Serious Adverse Reaction. |
| TDS | Three times a day |
| ULN | Upper limit of normal |

**Contents**

[1. Introduction 5](#_Toc164935291)

[1.1 Background and rationale 5](#_Toc164935292)

[1.2 Objectives 5](#_Toc164935293)

[2. Study methods 5](#_Toc164935294)

[2.1 Trial design 5](#_Toc164935295)

[2.2 Trial setting and patient population 5](#_Toc164935296)

[2.3 Definition of outcome measures 6](#_Toc164935297)

[2.4 Study assessments 8](#_Toc164935298)

[2.5 Sample size and power 10](#_Toc164935299)

[3. Statistical considerations 10](#_Toc164935300)

[3.1 Timing of analyses 10](#_Toc164935301)

[3.2 Analysis populations 10](#_Toc164935302)

[4. Study population 10](#_Toc164935303)

[4.1 Participant flow through trial 10](#_Toc164935304)

[4.1.1 Screening, eligibility and recruitment 10](#_Toc164935305)

[4.1.2 Protocol deviations 14](#_Toc164935306)

[4.1.3 Follow-Up 14](#_Toc164935307)

[4.2 Baseline characteristics 14](#_Toc164935308)

[4.3 Treatment adherence 15](#_Toc164935309)

[5. Analysis methods 16](#_Toc164935310)

[5.1 Analysis of primary outcome 16](#_Toc164935311)

[5.1.1 Intention to treat population 16](#_Toc164935312)

[5.2 Analysis of secondary outcomes 17](#_Toc164935313)

[5.2.1 Muscle Biopsy Outcomes. 17](#_Toc164935314)

[5.2.2 Whole Blood NAD Concentration 19](#_Toc164935315)

[5.2.3 MRI Outcome Measures 20](#_Toc164935316)

[5.2.4 Frailty and Physical activity 20](#_Toc164935317)

[5.2.5 Digital Measures of physical activity 7-day triaxial accelerometery (Axivity AX3) 21](#_Toc164935318)

[5.3 Missing data 22](#_Toc164935319)

[6. Safety 23](#_Toc164935320)

[6.1 Adverse events 23](#_Toc164935321)

[6.1.1 All adverse events 23](#_Toc164935322)

[6.1.2 All non-serious adverse events and adverse reactions 24](#_Toc164935323)

[6.2 Serious adverse events, serious adverse reactions, and suspected unexpected serious adverse reactions 24](#_Toc164935324)

[7. Verification of primary outcome 25](#_Toc164935325)

[8. statistical software 25](#_Toc164935326)

[references 25](#_Toc164935327)

# **Introduction**

## Background and rationale

The background and rationale for the study are set out in *Acipimox to improve muscle function and sarcopenia – a feasibility study protocol V2.0*

## Objectives

- - 1. **Primary objective**
- To test whether supplementation with acipimox improves skeletal muscle NAD+/NADH concentrations and ratio.
  - 1. **Secondary objectives**
- To test whether supplementation improves mitochondrial respiratory chain function.
- To test whether a precision target population (individuals with low skeletal muscle NAD levels on biopsy) can be identified by less invasive biomarkers.
- To assess candidate outcomes for a future randomised controlled trial
  - Phosphocreatine recovery rate measured by 31P MRS of the calf.
  - Changes in NAD(P)H levels by 31P MRS of the calf
  - Quantitative DIXON MRI of the calf muscles to assess for intra-muscular fat infiltration.
  - Diffusion weighted MRI to assess motor unit size via spontaneous muscle activity.
  - Short Physical Performance Battery
  - Maximal handgrip strength
  - 7-day triaxial accelerometry (Axivity AX6) to measure indices of physical activity, gait speed, variability, and postural control

# **Study methods**

## Trial design

This feasibility study is a single group, non-randomised, open-label uncontrolled trial. Participants with low grip strength and low walk speed will take acipimox 250mg two to three times a day for 17 days (+/- 3days). No placebo group will be used. Follow-up will be for a total of four weeks, with outcomes measured at baseline (days 0 and 7) and follow up (days 21 and 28) Study recruitment and follow-up process is shown in Figure 1. Schedule of assessments is shown in Table 1 Study assessments

##

## Trial setting and patient population

The trial is set in a single centre in Northeast England, with additional participant identification centres (PICs) in primary and secondary care.

**Inclusion Criteria**

- Age 65 years or over

- Low maximum handgrip strength (<16kg for women, <27kg for men) OR Prolonged 5 x sit to stand time (>15s) (Inability to complete five sit to stands will count as a prolonged sit to stand time)

- Walk speed <0.8 m/s on 4 metre walk test

**Exclusion Criteria**

General:

- Allergy to acipimox or other niacin-related products
- Allergy or intolerance of aspirin
- Any contraindication to taking aspirin
- Unable to give written informed consent
- Currently enrolled in another intervention study (observational studies are permitted)
- Currently participating in supervised exercise classes or physiotherapy
- Any progressive neurological or malignant condition with life expectancy <6 months

Safety of IMP and nIMP:

- Creatinine Clearance <45ml/min (by Cockcroft-Gault equation)
- Taking statin medication or fibrate medication
- Active peptic ulcer disease or dyspepsia

Safety of muscle biopsy and MRI:

- Platelets <100x10^9^/L at screening (contraindication to muscle biopsy)
- Presence of a bleeding diathesis or use of oral or parenteral anticoagulant medication
- Antiplatelet agents other than low dose (75mg once daily) aspirin
- Contraindications to MRI scanning (mild claustrophobia is not a contraindication)
- Allergy to local anaesthetic (lidocaine)
- Unable to palpate vastus lateralis muscle to enable biopsy localisation

Other causes of skeletal myopathy:

- Liver function tests (bilirubin, ALT, alkaline phosphatase) > 3x ULN
- Symptomatic (NYHA class II-IV) chronic heart failure (diagnosed according to European Society of Cardiology guidelines)
- Severe COPD (GOLD stage IV)
- Known myositis or other established myopathy
- Self-reported weight loss of >10% in last 6 months (to exclude significant cachexia)
- Known uncontrolled thyrotoxicosis
- 7.5mg/day or greater prednisolone use (or equivalent)

NB: Enrolling a patient onto the trial who does not meet the inclusion/exclusion criteria is considered a protocol waiver and is in breach of Regulation 29 (SI 2004/1031) of the Medicines for Human Use (Clinical Trials) Regulations 2004. Protocol waivers are not permitted.

## Definition of outcome measures

Outcome measures are shown below. See study protocol V2.0 for details of outcome measures.

- NAD+/NADH concentrations and ratio from skeletal muscle biopsy
- Skeletal muscle biopsy for:
  - Respiratory chain deficiency via histochemistry and quadruple immunofluorescence.
  - ATP/ADP concentrations and ratio
  - mtDNA copy number via quantitative PCR
- Whole blood NAD concentration
- Phosphocreatine recovery rate measured by 31P MRS of the calf
- Changes in NAD(P)H levels by 31P MRS of the calf
- Quantitative DIXON MRI of the calf muscles to assess for intra-muscular fat infiltration
- Diffusion weighted MRI to assess motor unit size via spontaneous muscle activity
- Short Physical Performance Battery
- Maximal handgrip strength
- 7-day triaxial accelerometery (Axivity AX6) to measure indices of physical activity, gait speed, variability, and postural control

**Conversion rate from screening to participant receiving IMP (recruitment)**

Data on the number of participants who are screened and eventually randomised will be captured.

**Retention rate of recruited participants at end of trial (4 months)**

Data on study withdrawal and lost to follow up status at the 28 days follow up visit will be captured.

*
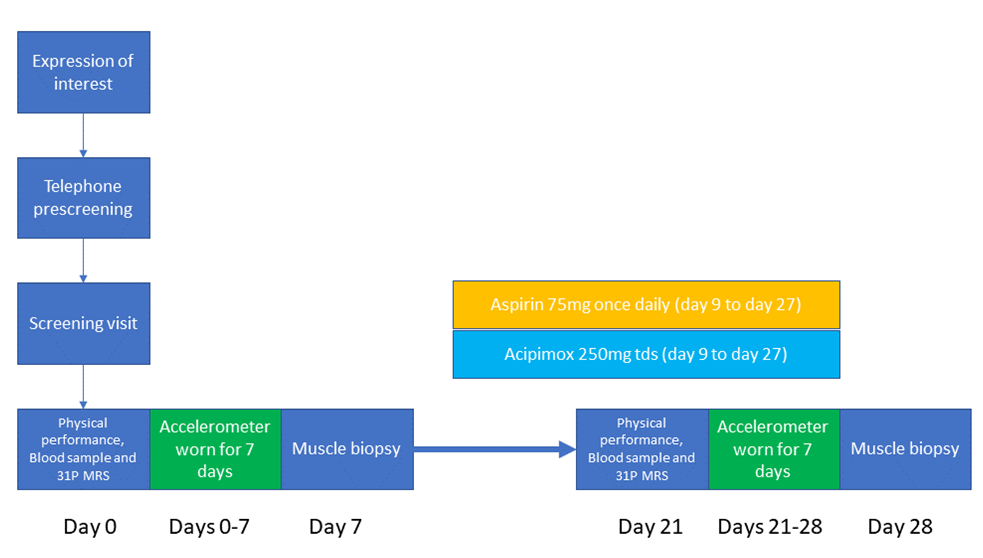
*

Figure 1 Study recruitment and follow-up process

## Study assessments

Table 1 Study assessments

|  | **Visit 1** | **Visit 2** | **Visit 3** | **Visit 4*** | **Visit 5** | **Visit 6** | **Visit 7** | **Visit 8** | **Visit 9** | **Visit 10** |
| --- | --- | --- | --- | --- | --- | --- | --- | --- | --- | --- |
| **Assessment** | **Pre-Screen** | **Screening** | **Baseline 1** | **Baseline 2** | **Phone call** | **Phone call** | **Follow up 1** | **Follow up 2** | **Phone call** | **Phone call** |
|  |  | **Day -28 to -1** | **Day 0** | **Day 7**  **(+/- 1 day)** | **Day 9 (+/- 1 day)** | **Day 14 (+/- 2 days)** | **Day 21 (+/- 3 days)** | **Day 28 (+/- 3 days)** | **Day 30 (+/- 3 days)** | **Day 37 (+/- 4 days)** |
| SARC-F questions | X |  |  |  |  |  |  |  |  |  |
| Demographics | X | X |  |  |  |  |  |  |  |  |
| Medical history | X | X |  |  |  |  |  |  |  |  |
| Concomitant medications | X | X | X | X |  |  | X | X |  |  |
| Informed written consent |  | X |  |  |  |  |  |  |  |  |
| Height |  | X |  |  |  |  |  |  |  |  |
| Weight |  | X |  |  |  |  |  |  |  |  |
| Bloods (U+Es, liver function tests) |  | X |  |  |  |  |  |  |  |  |
| Bloods (Full blood count) |  | X |  |  |  |  |  |  |  |  |
| Bloods (research bloods for NAD levels) |  |  | X |  |  |  | X |  |  |  |
| Frailty questions (activity, exhaustion, weight loss) |  |  | X |  |  |  | X |  |  |  |
| Muscle biopsy |  |  |  | X |  |  |  | X |  |  |
| MRI scanning |  |  | X |  |  |  | X |  |  |  |
| Accelerometer given out |  |  | X |  |  |  | X |  |  |  |
| Accelerometer returned |  |  |  | X |  |  |  | X |  |  |
| Medication dispensing |  |  |  | X |  |  |  |  |  |  |
| Instructions on taking medication |  |  |  |  | X |  |  |  | X |  |
| Medication adherence (collection/count) |  |  |  |  |  |  |  | X |  |  |
| Telephone check of wound healing |  |  |  |  | X | X |  |  | X | X |
| Adverse Events log completion |  | X | X | X | X | X | X | X | X | X |
| 4m walk speed |  | X |  |  |  |  |  |  |  |  |
| 5-times sit to stand |  | X |  |  |  |  |  |  |  |  |
| Grip strength |  | X | X |  |  |  | X |  |  |  |
| Short Physical Performance Battery |  |  | X |  |  |  | X |  |  |  |
| Eligibility confirmation |  | X |  |  |  |  |  |  |  |  |

##

## Sample size and power

As this is a feasibility study, few data exist on which to base a sample size calculation. The sample size has been selected to enable detection of a 1 SD change in measures of skeletal muscle NAD concentrations, ATP, skeletal muscle ATP concentrations or mitochondrial respiratory chain function. To detect this change with an alpha of 0.05 and 80% power requires 11 paired observations (using a paired t-test). We plan to recruit 16 participants to allow for dropout and non-completion of the course of medication.

# **Statistical considerations**

## Timing of analyses

The primary analysis will take place after the last patient recruited has completed their 28 day follow up visit (last patient, last visit). Final analyses will be carried out after all participants have been followed-up, all samples and imaging investigations have been analysed as set out in Protocol v2.0, all study data have been entered and database lock is complete.

## Analysis populations

**Intention to treat:** The primary analysis population is a modified intention-to-treat (mITT) population. This will consist of all participants with baseline and follow-up measures recruited regardless of whether they discontinued treatment.

**Per-protocol:** In addition, a per-protocol (PP) analysis will be performed, in participants with adherence to IMP ≥80%. Adherence will be assessed by Acipimox tablet count of returned medication bottles and calculated as number taken divided by the number expected to have been taken.

**Safety population:** A safety analysis will be performed examining AEs for all participants receiving at least one dose of IMP.

# **Study population**

## Participant flow through trial

Patient flow through the trial will be presented using a CONSORT diagram. Information will be provided on numbers and reasons (where appropriate) for: screened patients not being eligible; eligible patients not being allocated treatment; patients found to be ineligible after treatment initiation; patients deviating from treatment protocol (under-adherence defined as <80% of anticipated medication taken); temporary discontinuation of trial treatment; patients not evaluable for the primary endpoints; withdrawal from follow-up; death; withdrawal of consent and all protocol violations; and inclusion in analysis populations (mITT and PP).

## Screening, eligibility and recruitment

Screening and recruitment will be summarised. Reasons for ineligibility and reasons for eligible patients not being recruited will also be summarised in the CONSORT flow diagram. Screening data for those who pass and those who failed screening will be shown.

**Example table: Demographics, and measurements at screening**

|  | **Screen Pass** | **Screen Fail** |
| --- | --- | --- |
|  | **Total (n=)** | **Total (n=)** |
| **Demographic** |  |  |
| Age, years at baseline |  |  |
| Mean (sd) |  |  |
| Median (iqr) |  |  |
| Available data, n |  |  |
| Sex, n (%) |  |  |
| Male |  |  |
| Not stated |  |  |
| Available data, n |  |  |
| Height, cm at baseline |  |  |
| Mean (sd) |  |  |
| Median (iqr) |  |  |
| Available data, n |  |  |
| Weight, kg at baseline |  |  |
| Mean (sd) |  |  |
| Median (iqr) |  |  |
| Available data, n |  |  |
| Body mass index, cm/kg^2^ at baseline |  |  |
| Mean (sd) |  |  |
| Median (iqr) |  |  |
| Available data, n |  |  |
| Weight change in last 6 months to nearest kg |  |  |
| Mean (sd) |  |  |
| Median (iqr) |  |  |
| Available data, n |  |  |
| **Medical history (at screening)** |  |  |
| Any previous diagnosis of ischaemic heart disease, n (%) |  |  |
| Available data, n |  |  |
| Previous Stroke, n (%) |  |  |
| Available data, n |  |  |
| Chronic Heart Failure , n (%) |  |  |
| Available data, n |  |  |
| Diabetes Mellitus, n (%) |  |  |
| Available data, n |  |  |
| Atrial Fibrillation, n (%) |  |  |
| Available data, n |  |  |
| Hypothyroidism, n (%) |  |  |
| Available data, n |  |  |
| Hyperthyroidism, n (%) |  |  |
| Available data, n |  |  |
| Hypertension, n (%) |  |  |
| Available data, n |  |  |
| Dementia, n (%) |  |  |
| Available data, n |  |  |
| Anxiety, n (%) |  |  |
| Available data, n |  |  |
| Depression, n (%) |  |  |
| Available data, n |  |  |
| Parkinson’s Disease or Parkinson’s plus syndrome, n (%) |  |  |
| Available data, n |  |  |
| Chronic obstructive pulmonary disease, n (%) |  |  |
| Available data, n |  |  |
| Asthma, n (%) |  |  |
| Available data, n |  |  |
| Other lung disease, n (%) |  |  |
| Available data, n |  |  |
| Osteoarthritis, n (%) |  |  |
| Available data, n |  |  |
| Inflammatory arthropathy, n (%) |  |  |
| Available data, n |  |  |
| **Physical performance** |  |  |
| Maximum handgrip strength for men^#^ (kg) |  |  |
| Mean (sd) |  |  |
| Median (iqr) |  |  |
| Available data, n |  |  |
| Maximum handgrip strength for women^#^ (kg) |  |  |
| Mean (sd) |  |  |
| Median (iqr) |  |  |
| Available data, n |  |  |
| 4m walk speed (m/s) |  |  |
| Mean (sd) |  |  |
| Median (iqr) |  |  |
| Available data, n |  |  |
| 5x sit to stand time (s) |  |  |
| Mean (sd) |  |  |
| Median (iqr) |  |  |
| Available data, n |  |  |
| Number of medications  Mean (sd)  Median (iqr)  Available data, n |  |  |

**Example table: Results of blood tests where measured**

|  |  |
| --- | --- |
| Haemoglobin, g/L |  |
| Mean (sd) |  |
| Median (iqr) |  |
| Range |  |
| Available data, n |  |
| Platelets x10^9^/L |  |
| Mean (sd) |  |
| Median (iqr) |  |
| Range |  |
| Available data, n  White Cell Count x10^9^/L  Mean (sd)  Median (iqr)  Range  Available data, n |  |
| Sodium, mmol/L |  |
| Mean (sd) |  |
| Median (iqr) |  |
| Range |  |
| Available data, n |  |
| Potassium, mmol/L |  |
| Mean (sd) |  |
| Median (iqr) |  |
| Range |  |
| Available data, n |  |
| Urea, mmol/L |  |
| Mean (sd) |  |
| Median (iqr) |  |
| Range |  |
| Available data, n |  |
| Creatinine, µmol/L |  |
| Mean (sd) |  |
| Median (iqr) |  |
| Range  Available data, n |  |
| Bilirubin, µmol/L |  |
| Mean (sd) |  |
| Median (iqr) |  |
| Range  Available data, n |  |
| Alkaline phosphatase, U/L |  |
| Mean (sd) |  |
| Median (iqr) |  |
| Range |  |
| Available data, n |  |
| Alanine aminotransferase ALT, U/L |  |
| Mean (sd) |  |
| Median (iqr) |  |
| Range |  |
| Available data, n |  |
| Cockcroft-gault, ml/min |  |
| Mean (sd) |  |
| Median (iqr) |  |
| Range |  |
| Available data, n |  |

## Protocol deviations

Protocol deviations will be reported as shown below.

**Example table: Protocol deviations**

| Trial ID | Deviation type | Violation (Y/N) | Serious breach (Y/N) |
| --- | --- | --- | --- |
|  |  |  |  |
|  |  |  |  |
|  |  |  |  |

## Follow-Up

The number of patients lost to follow-up and/or withdrawn, with reasons where given, will be reported.

**Example table: withdrawls**

| Trial ID | Stage of withdrawal | Who led withdrawal | Reason | Description |
| --- | --- | --- | --- | --- |
|  | - Baseline Visit 1 MRI - Baseline Visit 2 Biopsy - Telephone call 1 - Telephone call 2 - Follow-up Visit 1 MRI - Follow-up Visit 2 Biopsy - Telephone call 3 - Telephone call 4 | - Participant requested - Investigator led |  |  |

## Baseline characteristics

Descriptive statistics will be used to report the baseline characteristics of the sample. Continuous data will be presented as mean (SD), median (IQR), and number with non-missing data. Categorical data will be presented as frequencies and percentages and number with non-missing data.

**Example table: Baseline measures**

| **Fried Frailty score, n(%)** |  |
| --- | --- |
| Non-frail (score of 0) |  |
| Pre-frail (1 – 2) |  |
| Frail status (≥3) |  |
| Mean (sd) |  |
| Median (iqr) |  |
| Available data, n |  |
| **Short Physical Performance Battery** |  |
| Mean (sd) |  |
| Median (iqr) |  |
| Available data, n |  |
| **Maximum handgrip strength for men^#^ (kg)** |  |
| Mean (sd) |  |
| Median (iqr) |  |
| Available data, n |  |
| **Maximum handgrip strength for women^#^ (kg)** |  |
| Mean (sd) |  |
| Median (iqr) |  |
| Available data, n |  |
| **4m walk speed (m/s)** |  |
| Mean (sd) |  |
| Median (iqr) |  |
| Available data, n |  |
| **5x sit to stand time (s)** |  |
| Mean (sd) |  |
| Median (iqr) |  |
| Available data, n |  |
| **Muscle Biopsy outcomes**  **NAD^+^ Concentration (uM in 0.5mg tissue)**  *Mean (sd)*  *Median (iqr)*  *Range*  **NADH Concentration (uM in 0.5mg tissue)**  *Mean (sd)*  *Median (iqr)*  *Range*  **NAD^+^/ NADH ratio**  *Mean (sd)*  *Median (iqr)*  *Range*  **ATP Concentration (nmol/mg)**  *Mean (sd)*  *Median (iqr)*  *Range*  **ADP Concentration (nmol/mg)**  *Mean (sd)*  *Median (iqr)*  *Range*  **ATP/ ADP ratio**  *Mean (sd)*  *Median (iqr)*  *Range*  **Mitochondrial DNA copy number (number of mitochondrial DNA molecules relative to 1 nuclear DNA)**  *Mean (sd)*  *Median (iqr)*  *Range*  **Complex 1 (NDUFB8)**   - Proportion normal (%) - Proportion abnormal (%)   **Complex 4 (Cox 1))**   - Proportion normal (%) - Proportion abnormal (%)   **COX/SDH (cytochrome c oxidase/succinate dehydrogenase) histochemistry**   - Proportion COX +ve (%) - Proportion intermediate or   negative (%) |  |
| **Blood NAD**  **NAD^+^ Concentration (uM)**  *Mean (sd)*  *Median (iqr)*  *Range*  **NADH Concentration (uM)**  *Mean (sd)*  *Median (iqr)*  *Range*  **NAD^+^ /NADH ratio**  *Mean (sd)*  *Median (iqr)*  *Range* |  |
| **MRI outcomes**  **Phosphocreatine recovery rate t½ Pcr (S)**  *Mean (sd)*  *Median (iqr)*  *Range*  **Percentage phosphocreatine depletion post exercise**  *Mean (sd)*  *Median (iqr)*  *Range* |  |
| **Accelerometery variables**  **Number of walking bouts a day**  *Mean (sd)*  *Median (iqr)*  *Range*  **Walking bout duration (seconds)**  *Mean (sd)*  *Median (iqr)*  *Range*  **Walking bout speed (m/s) in short bouts (10-30s)**  *Mean (sd)*  *Median (iqr)*  *Range* |  |

Data are n; %, mean (SD) or median (IQR); range, unless otherwise stated.

# Note the n here is different due to spilt by biological sex.

## Treatment adherence

Adherence is assessed by tablet count of returned medication bottles and calculated as number taken divided by the number expected to have been taken (expressed as a percentage).The number of tablets taken will be the number of trial medication dispensed (372 tablets) minus the number of tablets returned. The number of tablets expected to have been taken:

[Follow-up visit 2 date visit date) – (Medication start date)] x [ prescribed daily frequency (*BD or TDS*)]

The adherence assessment will take place only at the end of the trial, and low adherence will not result in withdrawal of participants from the trial analysis. If a participant has missing information on the returned tablet count we will report missing data.

Adherence will be summarised at the 4 month follow up with the median (IQR) percentage of medication taken. Reasons for premature discontinuation of allocated treatment are provided as a line listing.

**Example table: Summary of allocated treatment received.**

|  | Acipimox (n=) |
| --- | --- |
| Received at least one dose of Acipimox, n (%) |  |
| Treatment adherence, n (%) |  |
| Completed treatment (100%) |  |
| ≥80 to <100% |  |
| <80% |  |
| Temporary discontinuation of trial medication, n (%) |  |
| Discontinuation of trial medication, n (%) |  |
| Days from first to last dose, median (iqr) |  |
| % of total expected dose, median (iqr) |  |

Data are n; % or median (IQR); range, unless otherwise stated.

**Example table: Line listing of reasons for discontinuation of allocated trial medication**

| Trial ID | Date of discontinuation | Temporary? | Reason | Date of restart |
| --- | --- | --- | --- | --- |
|  |  |  | *Description* |  |

# **Analysis methods**

## Analysis of primary outcome

## Intention to treat population

The primary endpoint is change in NAD+/NADH concentrations and ratio from skeletal muscle biopsy following 19 (+/-3) days treatment with Acipimox. NAD+/NADH concentrations and ratio before and after treatment with Acipimox will be presented as mean (sd), median (iqr), and range where appropriate. Data will be plotted and inspected visually to determine normality Normally distributed data will be compared using a paired T test, non-parametric data will be compared using Wilcoxon signed rank test. Due to the paired nature of the analysis only participants who underwent testing at baseline and follow-up will be included.

We will present the mean (sd) of NAD+/NADH concentrations and ratio at baseline and at follow-up along with p-value.

**Example table: skeletal muscle biopsy NAD, NADH and NAD+/NADH ratio at baseline and follow up**

|  | Baseline | Follow up | Difference between baseline and Follow-up | P value |
| --- | --- | --- | --- | --- |
| **NAD^+^ Concentration (uM in 0.5mg tissue)** | *Mean (sd)*  *Median (iqr)*  *Range* | *Mean (sd)*  *Median (iqr)*  *Range* | *Mean (sd)*  *Median (iqr)* |  |
| **NADH Concentration (uM in 0.5mg tissue)** | *Mean (sd)*  *Median (iqr)*  *Range* | *Mean (sd)*  *Median (iqr)*  *Range* | *Mean (sd)*  *Median (iqr)* |  |
| **NAD^+^/ NADH ratio** | *Mean (sd)*  *Median (iqr)*  *Range* | *Mean (sd)*  *Median (iqr)*  *Range* | *Mean (sd)*  *Median (iqr)* |  |

A subgroup analysis will explore if there are differences between male and female participants.

## Analysis of secondary outcomes

All secondary analyses will be conducted in the ITT population. Data will be plotted and inspected visually to determine normality. Normally distributed data will be compared using a paired T test, non-parametric data will be compared using Wilcoxon signed rank test. Categorical data will be compared using McNemars test. Due to the paired nature of the analysis only participants who underwent testing at baseline and follow-up will be included.

## Muscle Biopsy Outcomes.

- ATP/ADP concentrations and ratio
- NAD+/ NADH
- mtDNA copy number via quantitative PCR
- Respiratory chain deficiency via histochemistry and quadruple immunofluorescence.

**Example table: skeletal muscle ATP, ADP and ATP/ ADP ratio at baseline and follow**

|  | Baseline | Follow- UP | Difference between baseline and follow-up | P value |
| --- | --- | --- | --- | --- |
| **ATP Concentration (nmol/mg)** | *Mean (sd)*  *Median (iqr)*  *Range* | *Mean (sd)*  *Median (iqr)*  *Range* | *Mean (sd)*  *Median (iqr)* |  |
| **ADP Concentration (nmol/mg)** | *Mean (sd)*  *Median (iqr)*  *Range* | *Mean (sd)*  *Median (iqr)*  *Range* | *Mean (sd)*  *Median (iqr)* |  |
| **ATP/ ADP ratio** | *Mean (sd)*  *Median (iqr)*  *Range* | *Mean (sd)*  *Median (iqr)*  *Range* | *Mean (sd)*  *Median (iqr)* |  |

**Example table: Mitochondrial DNA copy number at baseline and follow up**

|  | Baseline | Follow- UP | Difference between baseline and follow-up | P value |
| --- | --- | --- | --- | --- |
| **Mitochondrial DNA copy number (number of mitochondrial DNA molecules relative to 1 nuclear DNA)** | *Mean (sd)*  *Median (iqr)*  *Range* | *Mean (sd)*  *Median (iqr)*  *Range* | *Mean (sd)*  *Median (iqr)* |  |

**Example table: Proportion of fibres with normal complex 1 and complex IV at baseline and follow**

|  | Baseline | Follow- UP | P value |
| --- | --- | --- | --- |
| **Complex 1 (NDUFB8)**   - Proportion normal (%) - Proportion abnormal (%) |  |  |  |
| **Complex 4 (Cox 1))**   - Proportion normal (%) - Proportion abnormal (%) |  |  |  |
| **COX/SDH (cytochrome c oxidase/succinate dehydrogenase) histochemistry**   - Proportion COX +ve (%) - Proportion intermediate or   negative (%) |  |  |  |

## Whole Blood NAD Concentration

**Example table: blood NAD concentration at baseline and follow up**

|  | Baseline | Follow- Up | Difference between baseline and follow-up | P value |
| --- | --- | --- | --- | --- |
| **NAD^+^ Concentration (uM)** | *Mean (sd)*  *Median (iqr)*  *Range* | *Mean (sd)*  *Median (iqr)*  *Range* | *Mean (sd)*  *Median (iqr)* |  |
| **NADH Concentration (uM)** | *Mean (sd)*  *Median (iqr)*  *Range* | *Mean (sd)*  *Median (iqr)*  *Range* | *Mean (sd)*  *Median (iqr)* |  |
| **NAD^+^ /NADH ratio** | *Mean (sd)*  *Median (iqr)*  *Range* | *Mean (sd)*  *Median (iqr)*  *Range* | *Mean (sd)*  *Median (iqr)* |  |

## MRI Outcome Measures

#### PCr recovery rate,

#### Quantitative DIXON MRI of the calf muscles to assess for intra-muscular fat infiltration

#### Diffusion weighted MRI to assess motor unit size via spontaneous muscle activity

**Example table: PCr Recovery rate**

|  | Baseline | Follow- Up | Difference between baseline and follow-up | P |
| --- | --- | --- | --- | --- |
| Phosphocreatine recovery rate t½ Pcr (S) | *Mean (sd)*  *Median (iqr)*  *Range* | *Mean (sd)*  *Median (iqr)*  *Range* | *Mean (sd)*  *Median (iqr)* |  |
| Percentage phosphocreatine depletion post exercise | *Mean (sd)*  *Median (iqr)*  *Range* | *Mean (sd)*  *Median (iqr)*  *Range* | *Mean (sd)*  *Median (iqr)* |  |

## Frailty and Physical activity

#### Frailty

Frailty will be assessed using the fried frailty score. Number of participants classed as robust, prefrail and frail at baseline and follow up will be shown.

| Fried Frailty Criteria | Baseline | Follow- UP |
| --- | --- | --- |
| - Robust (n) - Pre-Frail (n) - Frail (n) |  |  |

#### Maximum Hand Grip Strength

Summary statistics for maximal hand grip strength and SPBB will be presented as mean (sd), median (iqr), and range or n (%), where appropriate. Normally distributed data will be compared using a paired T test, non-parametric data will be compared using Wilcoxon Matched pairs test. Hand grip strength data will be presented separately for men and women.

**Example table: Maximum grip strength and SPBB at baseline and follow up**

|  | Baseline | Follow- UP | Difference between baseline and follow-up | P value |
| --- | --- | --- | --- | --- |
| - SPPB   - Available data, n | *Mean (sd)*  *Median (iqr)*  *Range* | *Mean (sd)*  *Median (iqr)*  *Range* | *Mean (sd)*  *Median (iqr)* |  |
| - 4M walk speed | *Mean (sd)*  *Median (iqr)*  *Range* | *Mean (sd)*  *Median (iqr)*  *Range* | *Mean (sd)*  *Median (iqr)* |  |
| - Maximum handgrip strength for men (kg)   - Available data, n - Maximum handgrip strength for women (kg)   - Available data, n | *Mean (sd)*  *Median (iqr)*  *Range*  *Mean (sd)*  *Median (iqr)*  *Range* | *Mean (sd)*  *Median (iqr)*  *Range*  *Mean (sd)*  *Median (iqr)*  *Range* | *Mean (sd)*  *Median (iqr)*  *Mean (sd)*  *Median (iqr)* |  |

#### Sit to stand

Repeated Sit to Stand test is a points-based tests. Summary statistics will be presented as the number and percentage of participants able to complete each stage of the test. Time to complete test will be presented as mean (sd), median (iqr), and range where appropriate. Normally distributed data will be compared using a paired T test, non-parametric data will be compared using Wilcoxon Matched pairs test

**Example table: Sit to stand at baseline and follow up**

|  | Baseline | Follow- UP | Difference between baseline and follow-up | P value |
| --- | --- | --- | --- | --- |
| - 5x sit to stand time (s)   - Able to complete without using hand,   - Able to complete in 60 seconds | n (%)  n (%) | n (%)  n (%) |  |  |
| - Time to complete 5 stands | *Mean (sd)*  *Median (iqr)*  *Range* | *Mean (sd)*  *Median (iqr)*  *Range* | *Mean (sd) Median (iqr)* |  |

## Digital Measures of physical activity 7-day triaxial accelerometery (Axivity AX3)

#### Number of walking bouts / day

- Walking bout duration
- Walking speed in short bouts (10-3s)

|  | Baseline | Follow- UP | Difference between baseline and follow-up | P value |
| --- | --- | --- | --- | --- |
| - Number of walking bouts per day | *Mean (sd)*  *Median (iqr)*  *Range* | *Mean (sd)*  *Median (iqr)*  *Range* | *Mean (sd)*  *Median (iqr)* |  |
| - Walking bout duration (seconds) | *Mean (sd)*  *Median (iqr)*  *Range* | *Mean (sd)*  *Median (iqr)*  *Range* | *Mean (sd)*  *Median (iqr)* |  |
| - Walking bout speed (m/s) in short bouts (10-30s) | *Mean (sd)*  *Median (iqr)*  *Range* | *Mean (sd)*  *Median (iqr)*  *Range* | *Mean (sd)*  *Median (iqr)* |  |

## Missing data

**Missing data presentation**

We will present missing data with tables describing the frequency and percentage of available data of each primary and secondary outcome, stratified by treatment arm.

**Example table: Available data rate for primary and secondary outcomes**

|  | Baseline  N (%) | Follow up  N (%) |
| --- | --- | --- |
| NAD+ Concentration |  |  |
| NADH Concentration |  |  |
| NAD/ NADH ratio |  |  |
| Respiratory chain deficiency via histochemistry and quadruple immunofluorescence. |  |  |
| ATP/ADP concentrations and ratio |  |  |
| mtDNA copy number via quantitative PCR |  |  |
| Whole Blood NAD Concentration |  |  |
| Changes in NAD(P)H levels by 31P MRS of the calf |  |  |
| Maximum Hand Grip Strength |  |  |
| SBPP |  |  |
| 5 sit to stands |  |  |
| 5 Sit to stand time |  |  |
| Activity monitor data |  |  |

**Missing data imputation**

No imputation will be made for missing data.

#

# **Safety**

## Adverse events

Safety data will be coded according to the Medical Dictionary for Regulatory Activities (MedDRA) for all AEs and SAEs. The Chief Investigator and Deputy Chief Investigator are responsible for reviewing and assigning MedDRA codes. MedDRA® the Medical Dictionary for Regulatory Activities terminology is the international medical terminology developed under the auspices of the International Council for Harmonisation of Technical Requirements for Pharmaceuticals for Human Use (ICH) [1]. Under MedDRA coding, each AE is standardised to a ‘preferred term’ which is “a distinct descriptor (single medical concept) for a symptom, sign, disease diagnosis, therapeutic indication, investigation, surgical or medical procedure, and medical social or family history characteristic.” Each adverse event is also categorised under a larger “system organ class” (SOC) umbrella term “which are groupings by aetiology (e.g. Infections and infestations), manifestation site (e.g. Gastrointestinal disorders) or purpose (e.g. Surgical and medical procedures). In addition, there is a SOC to contain issues pertaining to products and one to contain social circumstances” [1].

All non-serious AEs, ARs, SAEs, SARs, and SUSARs will be summarised as follows according to published guidelines and recommendations [2, 3]. No statistical analysis is planned for safety data.

## All adverse events

**Example Table: Number of adverse events per participant and frequency of participants with adverse events**

|  | Total (n=) |
| --- | --- |
| Number of adverse events per participant |  |
| Mean (SD) |  |
| Median (IQR) |  |
| Range |  |
| Number of participants with at least one adverse event, n (%) |  |
| Number of participants with at least one adverse reaction, n (%) |  |
| Number of participants with at least one serious adverse event, n (%) |  |
| Number of participants with at least one serious adverse reaction, n (%) |  |
| Number of participants with at least one SUSAR, n (%) |  |

**Example Table: Frequency and percentage of the worst severity reported by each participant.**

| Worst severity reported, n(%) | Total (n=) |
| --- | --- |
|  |  |
| No adverse event |  |
| Mild |  |
| Moderate |  |
| Severe |  |
| Life threatening |  |
| Death |  |
| Missing |  |

**Example Table: Frequency of participants affected by each reported adverse event.**

|  | Total (n=) |
| --- | --- |
| *System organ class, n* |  |
| *Preferred term A1*, n |  |
| *Preferred term A2*, n |  |

**Example Table: Number of adverse events related to study procedures.**

|  | Total (n=) |
| --- | --- |
| Number AE related to study procedure |  |
| Number of AE related to IMP |  |
| Number of AE unrelated to IMP |  |

## All non-serious adverse events and adverse reactions

**Example Table: Frequency of participants affected by each reported non-serious adverse event**

|  | Total (n=) |
| --- | --- |
| *Preferred term A1*, n |  |
| *Preferred term A2*, n |  |

## Serious adverse events, serious adverse reactions, and suspected unexpected serious adverse reactions

Separate line listings for SAEs which are reportable and not reported to sponsor, SARs, and SUSARs will be reported with information on, date of first IMP dose, date and time of onset and resolution, description of event, severity, seriousness of the event, action taken, and whether they were expected. The number of SAEs/SAR/SUSAR and the number of patients reporting at least one SAE/SAR/SUSAR will be reported in each group.

**Example Table: Line listing of SAEs reportable to sponsor, SARs, and SUSARs**

| **SAE no.** | **First dose date** | **AE Start date** | **Resolution date** | **Description** | **Severity^A^** | **Relationship to IMP^B^** | **Action taken^C^** | **Patient outcome^D^** |
| --- | --- | --- | --- | --- | --- | --- | --- | --- |
|  |  |  |  |  |  |  |  |  |

^A^Mild/Moderate/Severe/Life threatening/Death

^B^Unrelated/Unlikely to be related/Possibly related/Probably related/Definitely related

^C^Dose reduced/IMP withdrawn/None

^D^Resolved/Ongoing

**Example Table: Frequency and percentage of participants affected by all SAEs, SARs, and SUSARs**

|  | **Total (n=)** | |
| --- | --- | --- |
|  | **N** | **%** |
| Preferred term 1 |  |  |
| Preferred term 2 |  |  |

# **Verification of primary outcome**

During the final analysis, the computation and primary analysis of the primary outcome at baseline and four month follow up will be performed by deputy chief investigator and quality checked by the chief investigator. Quality checking of secondary outcomes and analyses will be performed by the deputy CI and CI

# **statistical software**

Data will be exported from the trial database as a Microsoft Excel file. Statistical analyses will be carried out by the deputy chief investigator using SPSS version 29.0.1.0.(171) IBM. All output will be stored in the Acipimox folder on the AGE RDW server.

# **references**

The content of this analysis plan follows, where appropriate, the International Conference on Harmonisation (ICH) Topic E3, Guideline for Structure and Content of Clinical Study Reports [5].

1. MedDRA. *MedDRA Hierarchy*. n.d.; Available from: <https://www.meddra.org/how-to-use/basics/hierarchy>.

2. Lineberry, N., et al., *Recommendations to improve adverse event reporting in clinical trial publications: a joint pharmaceutical industry/journal editor perspective.* BMJ, 2016. **355**: p. i5078.

3. International Council for Harmonisation of Technical Requirements for Registration of Pharmaceuticals for Human Use, *ICH E3 Structure and content of clinical study reports (CPMP/ICH/137/95)*. 1996, European Medicines Agency: London, UK.
